# Isolated domains of recombinant human apo-metallothionein 1A are folded at neutral pH: a denaturant and heat-induced unfolding study using ESI-MS

**DOI:** 10.1042/BSR20180592

**Published:** 2018-07-18

**Authors:** Gordon W. Irvine, Natalie Korkola, Martin J. Stillman

**Affiliations:** Department of Chemistry, The University of Western Ontario, London, ON, N6A 5B7

**Keywords:** denaturant, ESI-MS, metallothionein, metal-induced folding, protein conformation

## Abstract

Metallothioneins (MTs) are characterized by their high metal loading capacity, small molecular weight, and abundant cysteine residues. It has long been thought that metal-free, or apo-MT peptides were unstructured and only adopted as a distinct conformation upon forming the metal clusters, described as metal-induced folding. More recent studies have suggested that the presence of a globular, yet loosely defined structure actually exists that can be disrupted or unfolded. Residue modification and ion-mobility ESI (IM-ESI)-MS have been used to examine this unusual unfolding process. The structure of apo-MT plays a critical role as the starting point in the flexible metalation pathways that can accommodate numerous soft metals. ESI-MS measurements of the product species formed following the cysteine alkylation of the isolated domain fragments of recombinant human apo-MT 1A with *n*-ethylmaleimide (NEM) were used in the present study to monitor the denaturant- and heat-induced unfolding at physiological pH. The results indicate that these apo-MT fragments adopt distinct structures at neutral pH that react co-operatively with NEM when folded and non-cooperatively when heated or exposed to high concentrations of the denaturant guanidinium chloride (GdmCl). From these studies, we can conclude that at neutral pH, the domain fragments are folded into globular structures where some of the free cysteine residues are buried within the core and are stabilized by hydrogen bonds. Metalation therefore, must take place from the folded conformation.

## Introduction

Metallothioneins (MTs) are a family of metal-binding proteins found throughout the nature that are primarily involved in regulating Zn and Cu homeostasis [1,2]. These promiscuous proteins have been isolated from a wide range of biological sources containing numerous d-block metals [3–5] and have been shown to bind a number of exotic metals, including Rh [6], Pt [7,8], U [9], and As [10] amongst others, in *in vitro* studies. This flexibility in metal co-ordination is thought to originate from the loosely defined structure of apo-MT peptide chain, allowing the backbone and abundant cysteine residues (20 cysteines making approximately 30% of all the residues in MT, with no disulphide bond formation) to conformationally adjust in order to bind the metals in a variety of co-ordination modes [11]. The flexible structure of metal-free or apo-MT led to the assumption that the protein was unstructured or randomly oriented until metals were introduced [12]. This assumption was not without merit, as spectroscopic studies showed a distinct lack of secondary structure in apo-MT [13], and even in partially metalated MTs under certain conditions [14]. Simply, well-defined structure in MTs can be summarized as the result of metal-induced folding [12].

The introduction of ESI-MS was significant for the study of MTs because it allowed detailed investigation of the binding pathways of the multiple spectroscopically silent metals such as zinc and copper(I), that bind to MTs, revealing both the exact stoichiometries as metals bound and then elucidation of complex binding pathways [15–17]. The ESI-mass spectral data have been validated in the terms of accuracy in determining the relative concentrations of MT species with different metal loadings by comparison with atomic absorption data [18]. That the relative abundance can be extracted with confidence from the ESI-MS experiment indicates that the structural changes upon metalation do not result in radically different ionization efficiencies between species. Conclusion from these many reported methods, including techniques such as charge state analysis [19], ion-mobility (IM) MS [20], and residue modification coupled with ESI-MS [21], and LC-MS [22], cast doubt on the model of a completely unstructured apo-MT at physiological pH. However, these mass spectral studies were not the first to suggest a compact and globular structure, as theoretical calculations predicted that the cysteine residues in the apo-MTs were buried and not freely accessible to the solvent [23] and FRET studies [24,25] of recombinant MTs in the early 2000s showed that there was also negligible volume change in the protein upon metalation.

In particular, residue modification of the reactive cysteinyl thiols has proven to be effective in probing the relative accessibility of individual residues and the overall folded state of apo- and partially metalated MTs. In addition to probing structure, residue modification coupled with MS/MS has been used to identify the binding sites and monitor metalation/demetalation of MT with Zn, Cd [26], and As [27].

Recently, our group and others have demonstrated that the reaction with cysteine alkylation agents, such as *n*-ethylmaleimide (NEM), *p*-benzoquinone, and iodoacetamide can be used to determine the folded state of apo-MT under various conditions [21]. The most common denaturants used in biochemical unfolding studies are acidity and guanidinium chloride (GdmCl). The change in the folded state of most proteins during pH titrations can easily be monitored by CD spectroscopy or changes in ESI-MS charge states. However, for MT, the CD spectra are unchanged upon denaturation as the native form contains no secondary structural elements, and the protein is small enough that denaturing does not cause a dramatic shift in the charge state distribution. In addition, while apo-MT exhibits different reaction profiles at neutral and low pH; when exposed to large cysteine modifying agents, the alkylation reaction can be influenced by the presence of H^+^ and it is unclear whether the reaction profile divergence is solely due to unfolding. Typically, thermal denaturation has been used to demonstrate the chemical-free unfolding but there have been no recent reports of thermal denaturation of MTs to our knowledge [28].

In the present study, we probe the conformations of the isolated α- and β-domain fragments of recombinant human MT1a using cysteine alkylation coupled with ESI-MS in the presence of GdmCl, a well-known denaturant, and extreme heat (94°C). These methods of unfolding do not rely on acidification (high (H^+^)) but serve to disrupt H-bonding within the protein, which has been predicted to stabilize the apo-MT structure at physiologically reasonable pH [29].

## Materials and methods

### Reagents

All chemicals were used without further purification. Tris(2-carboxyethyl)phosphine (TCEP), NEM, cadmium sulphate, GdmCl, and ammonium formate were purchased from Sigma–Aldrich (St. Louis, MO). The isolated α- and β-domain fragments were overexpressed recombinantly using procedures described in detail elsewhere with no modification [30].

### Protein preparation

The protein was prepared recombinantly and purified as the S-tag peptides Cd_4_αMT (MGKAAAAC CSCCPMSCAK CAQGCVCKGA SEKCSCCKKA AAA) and Cd_3_βMT (MGKAAAACSC ATGGSCTCTG SCKCKECKCN SCKKAAAA) from *Escherichia coli* BL21(DE3) cells induced by IPTG. Cadmium was added to the growth medium because of its strong binding to the large amount of MT produced by the bacteria, preventing oxidation during purification. In addition, the strong ligand-to-metal charge transfer band at 250 nm arising from the Cys–Cd bond allowed for the detection of the protein during HPLC purification (Lifemate 3000 UHPLC, Dionex) and to determine the concentrations using the molar extinction coefficients (αε_250_ = 45000 M^−1^.cm^−1^, βε_250_ = 36000 M^−1^.cm^−1^) [31]. The S-tag was cleaved using thrombin as previously described to provide the sequence shown above. The protein samples were stored at −20°C prior to use.

### Apo-MT preparation

The protein samples were thawed under vacuum and buffer exchanged under Ar gas with Ar bubbled buffer solutions (10 mM ammonium formate). First, the sample was buffer exchanged with a 10 mM ammonium formate solution pH adjusted with formic acid to pH 2.8 to completely demetalate MT. The exchange was performed with 3-kDa MWCO centrifugal filters (Millipore, U.S.A.) at 3500 r.p.m. for 30 min per cycle for four cycles. Then, the demetalated protein was buffer exchanged with a degassed 10 mM ammonium formate solution containing 1 mM TCEP and adjusted to pH 7.4.

Metal-free MTs are quite susceptible to oxidation, therefore, great care was taken to exclude oxygen from the solutions. Oxidation can be observed in the mass spectra by a decreased mass of 2 Da for every Cys–Cys disulphide that formed. This was not observed in the spectra presented herein. In addition, the disulphide was unreactive toward NEM, and results in incompletely modified species, even when excess NEM was added. Again, this was not observed in the titrations described in the present paper, but an example of a partially incomplete reaction due to oxidation can be found in the literature [21].

### Denaturation

Once the protein solutions were prepared at neutral pH, they were mixed in various ratios with a pH adjusted 8 M GdmCl solution to obtain final GdmCl concentrations of 0.1–6 M. To these solutions, 3–5 molar equivalents of NEM were added and left to equilibrate for 5 min. Then, the solutions were buffer exchanged using centrifugal filters in the same manner as in the apo-MT preparation section to remove the GdmCl salt for subsequent mass spectral analysis.

For heat denaturation, 20 µM apo-βMT solution was added to a 2-ml vial, sealed tightly with parafilm, and placed in a water bath set at 94°C and with the temperature separately monitored with a digital thermometer. The protein solution was allowed to equilibrate in the water bath along with a vial containing the NEM solution for 10 min. Approximately 3 molar equivalents of hot NEM were added to the vial containing the apo-MT and allowed to react for 5 min. The vial was then removed from the bath, allowed to cool, and subsequently analyzed by ESI-MS.

### MS

The ESI mass spectral data were collected on a Bruker Micro-TOF II (Bruker Daltonics, Toronto, ON) operated in the positive ionization mode and calibrated with NaI. The desalted protein solutions were introduced into the MS at a rate of 10 µl/min. Settings: scan = 500–3000 m/z; rolling average = 2; nebulizer = 2 bar; dry gas = 180°C @ 6.0 l/min; capillary = 4000 V; end plate offset = −500 V; capillary exit = 175 V; Skimmer 1 = 30.0 V; Skimmer 2 = 23.5 V; Hexapole RF = 800 V. The spectra were averaged over 1–2 min collection time and deconvoluted using the maximum entropy (Max Ent) application of the Bruker Compass Data Analysis software package.

### Molecular dynamics calculations

Molecular modeling calculations were performed using Scigress version 3.0.0 (Fujitsu Poland Ltd.). The structure for human β (2MHU) domain of MT was extracted from the Protein Data Bank [32] and modified to the recombinant sequence using the sequence builder of the Scigress software program. The protonation state of the amino acid residues reflected a physiological pH of approximately 7. The adjusted protein constructs were energy minimized to achieve an optimized structure using the conjugate gradient method. The dielectric for water was set to 78 and the van der Waals cut-off distance was set to 9 Å at 300 K.

## Results

GdmCl was used to denature the apo-MT fragments as in traditional denaturing experiments, but the extremely high concentrations required are not compatible with ESI-MS. As remedy for this problem, GdmCl was removed by buffer exchange prior to measurement following the alkylation reaction. Since the alkylation reaction is irreversible, the post-reaction processing had no effect on the reaction profile or the distribution of alkylated species. The folded protein generally reacts in a co-operative manner due to the alkylation-induced unfolding of the Cys-rich protein, as described in detail in previous reports [21,33]. The uniqueness of this process will be described in detail in the ‘Discussion’ section and can be visualized in Figure 4. When the protein is unfolded, all cysteine residues are equally accessible and alkylation occurs in a stochastic manner yielding a binomial distribution of alkylated species that centers on the molar equivalent ratio of the alkylating agent added extending either side representing the populations of peptide more or less fractionally alkylated.

**Figure 1:**
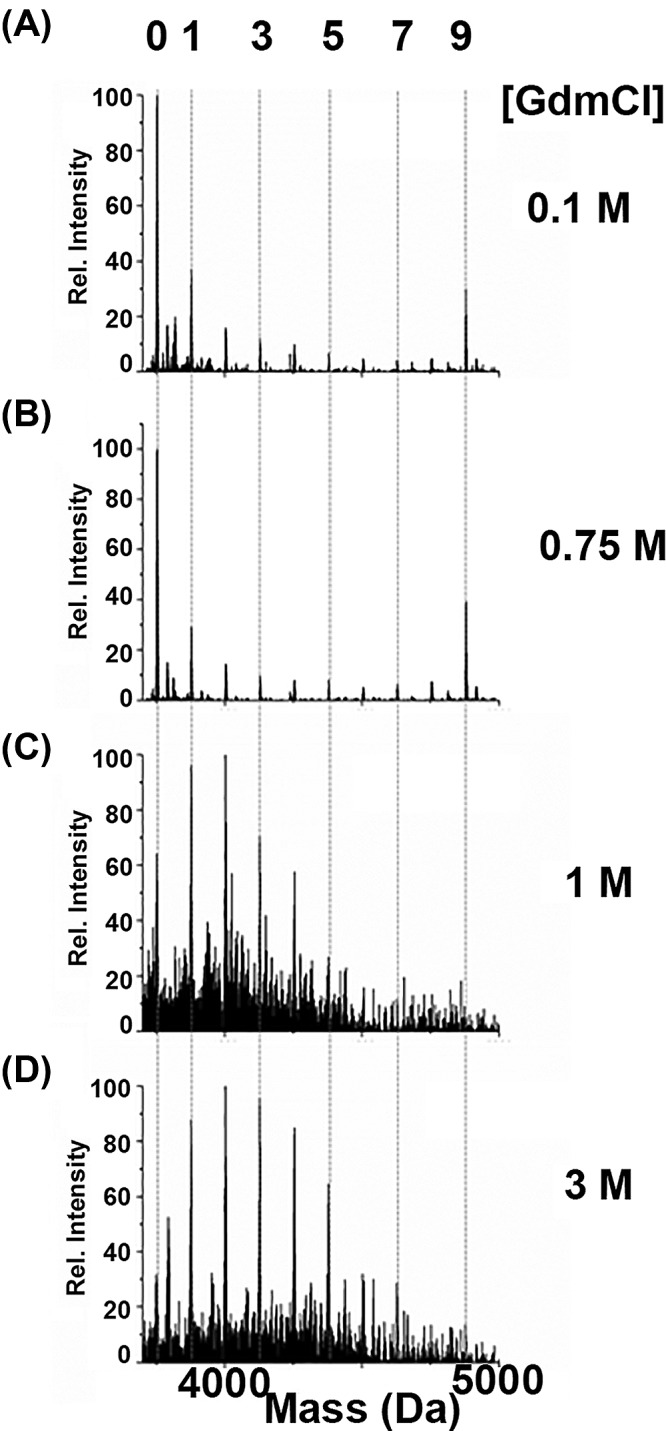
Representative deconvoluted ESI-mass spectra of the alkylated apo-βMT with approximately 3 mol. eq. NEM at pH 7.4 in the presence of increasing concentrations of GdmCl The deconvoluted mass spectra of apo-βMT reacted in aqueous solutions of (**A**) 0.1, (**B**), 0.75, (**C**) 1, and (**D**) 3 M GdmCl are presented. The reaction profiles in (A) and (B) are representative of the folded protein and those of (C) and (D) are typical of the unfolded protein. The numbers on the top *x*-axis indicate the number of NEM-modified cysteines and the dashed lines indicate the corresponding mass of the modified species and help guide the eye.

Figure 1 shows representative deconvoluted mass spectra of the alkylated apo-βMT with NEM in the presence of increasing concentrations of GdmCl. The numbers along the top *x*-axis in the figure corresponding to the number of NEM-modified cysteine residues represented by the species measured for the addition of 3 mol. eq. the alkylating agent, which can be up to 9 in the isolated β-domain fragment and 11 in the isolated α-domain fragment as a function of extent of unfolding. A distinct co-operative reaction profile is observed in Figure 1A,B with the lower concentrations of GdmCl (0.1 and 0.75 M, respectively), suggesting that the apo-protein is folded. Upon increasing the GdmCl concentration to 1 M or higher (Figure 1C,D), the reaction profile now shows a normal or binomial distribution of the alkylated species, providing evidence that the apo-protein is now unfolded. These results indicate that apo-βMT unfolds between >0.75 and ≤1 M GdmCl.

**Figure 2:**
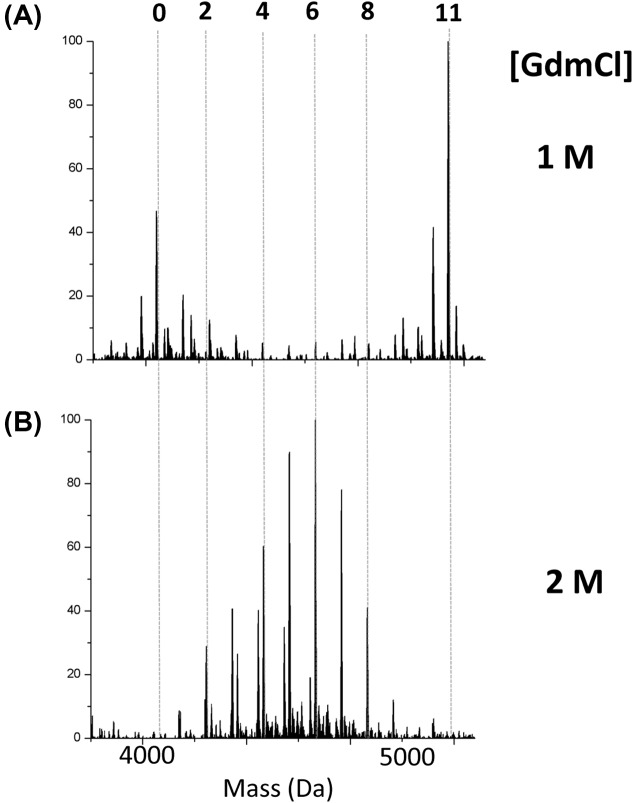
Representative deconvoluted ESI-mass spectra of the alkylated apo-αMT with approximately 6 mol. eq. NEM at pH 7.4 in the presence of various concentrations of GdmCl Deconvoluted mass spectra of apo-αMT reacted with NEM in the presence of (**A**) 1 and (**B**) 2 M GdmCl clearly show the folded (A) and unfolded (B) reaction profile. The numbers on the top *x*-axis of the figure indicate the number of NEM modifications of the cysteine residues and the dashed lines are drawn to indicate the mass and help guide the eye for comparison between the two spectra.

Similar to the β-domain fragment, the isolated α-domain fragment was tested for its folded status under increasing concentrations of GdmCl using alkylation with NEM. The α-domain fragment has 11 cysteine residues, that is why the top *x*-axis numbering in Figure 2 differs from that of Figure 1. Figure 2 shows representative deconvoluted mass spectra of the alkylated apo-αMT with Figure 2A exhibiting a co-operative reaction profile at 1 M GdmCl and Figure 2B showing a stochastic reaction profile at 2 M GdmCl. These results indicate that apo-αMT unfolds between >1 and ≤2 M GdmCl; which is higher than that of the β-domain fragment. These results provide evidence that the folded state of the α-domain fragment is more stable than that of the β-domain fragment, as it is less easily disrupted by the denaturant requiring a greater concentration to yield the binomial distribution (compare the distributions of alkylated species in Figure 1C at 1 M with Figure 2A at 1 M).

**Figure 3:**
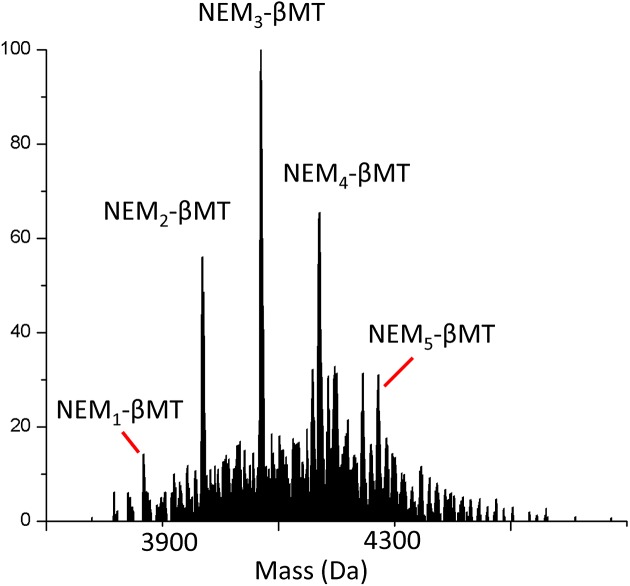
Representative deconvoluted mass spectrum of apo-βMT modified with approximately 3 mol. eq. of NEM at 94°C

A second commonly used denaturant method is heat. High temperatures were used to denature the apo-protein as it required no change to the solution composition as with denaturing using pH or denaturant. Figure 3 shows the representative mass spectra of apo-βMT alkylated with NEM at 94°C. This temperature was chosen as it was used in MT isolation procedures from rabbit liver and other biological sources before recombinant technology was available. This temperature typically causes larger proteins to precipitate, but unusually, MT remains in solution, although its folded state was unknown at this temperature. The mass spectrum in Figure 3 clearly shows a normal distribution of modified species, indicating that apo-βMT is unfolded at high temperatures. The results in Figure 3 clearly demonstrate that cysteine alkylation techniques can be used for all kinds of denaturing techniques and are largely unaffected by solution composition. Comparing the alkylation patterns in Figures 1–3 under denaturing conditions (3, 2 M GdmCl and 94°C, respectively), all exhibit a normal pattern of a binomial distribution of modified species, indicating an unfolded protein with unhindered access to cysteinyl thiols.

## Discussion

Until recently, mammalian apo-MTs were thought to be unstructured allowing the 20 Cysteines to conformationally adjust to the co-ordination preferences of the various metal ions present in the cellular milieu. However, molecular modeling and mass spectrometric methods have cast doubt on this hypothesis. IM-MS studies demonstrated that the presence of multiple conformers of the apo-protein in the gas phase [20] and kinetic studies showed that MT binds Cd^2+^ more rapidly under native conditions where the protein is folded [33]. This is counterintuitive as it might be expected that an open and unfolded structure would more rapidly bind metals in solution due to reduced steric hindrance compared with a folded protein where the potentially co-ordinating cysteines are buried. Our reasoning for this statement is that when one considers the demand for four terminal cysteinyl thiolates to form the [Zn(Scys)4]^2−^structures for the first Zn(II) ions bound, it would seem that the unfolded peptdie with the cysteines exposed would offer an easier route than the folded and compact conformation. Recalling that the metal-free or apo structure in MT is not specifically set up as a metal binding site as the metal binding site structure does not exist without metals in MTs, unlike almost all other metalloproteins; it is possible that this design allows for the binding of the tetrahedrally co-ordinating Zn(II) simultaneously with the trigonally co-ordinating Cu(I) in the same peptide. Thus, it is proposed that the structure of apo-MT provides a template for rapid metal binding, but not strictly defined binding sites [33]. This was controversial, as it is likely that this description is applicable to the wide variety of MTs present in worms, plants, mammals etc. In a recent study comparing human isoforms MT1 and 2, it was shown that Zn binding properties were very similar [34], so general conclusions are drawn regarding a single isoform of MT likely remain valid, but we are careful in generalizing the results drawn in the present study to all MTs at this time.

### Probing the structure of apo-MTs

Working with apo-MTs presents many difficulties in that the protein is air sensitive, so extra precautions must be taken. In addition, it cannot be quickly characterized by optical methods because mammalian MTs lack aromatic amino acids and secondary structure, precluding UV-Vis absorption and CD spectroscopic analyses. In NMR studies, only MT species where the stable cluster structure was formed were able to be observed, and even partially metalated intermediates resulted in broad indistinct peaks [14]. CD spectroscopy has been used to probe the structure of metalated MTs [35] and is used extensively to monitor folding of larger and more ordered proteins [36,37]. Even some MS-based techniques such as hydrogen–deuterium exchange require the formation of extensive hydrogen-bonding networks, usually in the form of secondary structural elements [38]. Thus, the profiling of the stepwise alkylation of the cysteinyl thiols with ESI-MS is one of the few suitable experimental methods for determining the structure of apo-MT. Here, it was used to distinguish between extended or unfolded conformations in which all cysteine residues are equally accessible and reactive, and globular or folded conformations in which a fraction of the cysteine residues are buried.

**Figure 4:**
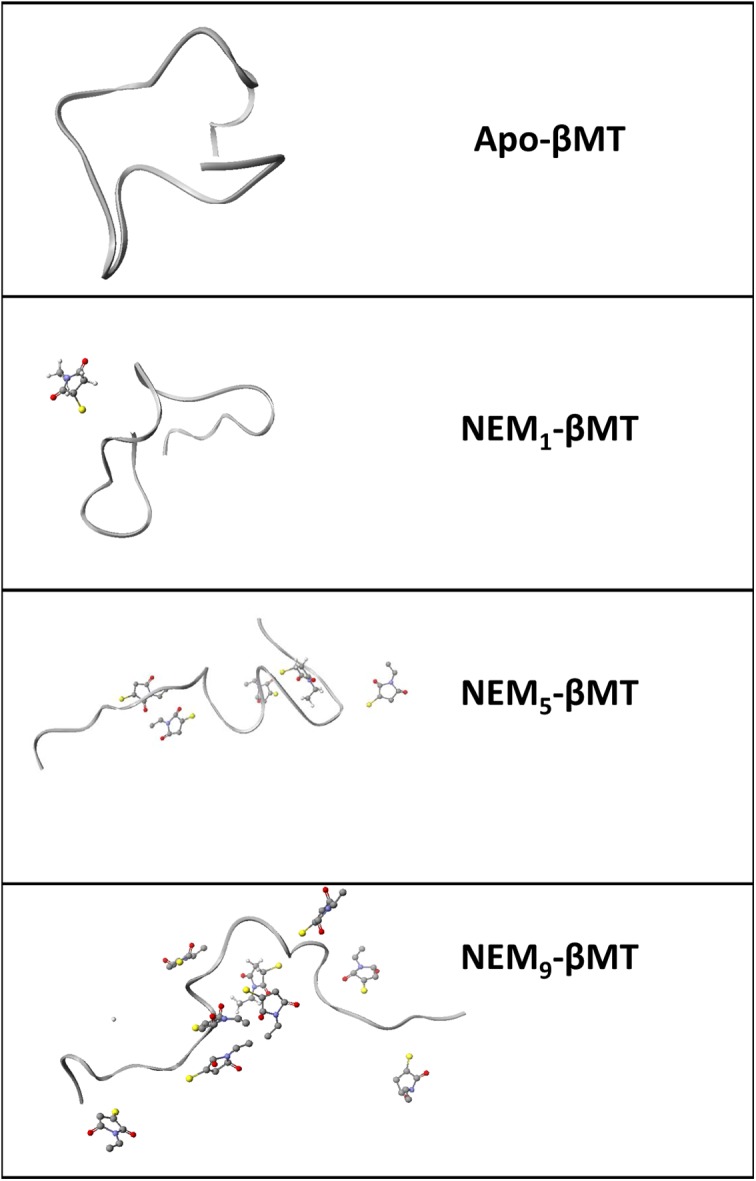
Ribbon visualization from MM/MD calculated structures of apo- and NEM_x_-βMT models at 300 K

In the folded state, the cysteines in apo-MT are unequally accessible and the first alkylation event triggers the adoption of a new conformation, which is more open, exposing previously buried cysteines. The alkylated proteins with exposed cysteines are then more likely to further react with NEM, providing a mechanism for the co-operative reaction that can be seen in the mass spectra in Figures 1 and 2. This process can be observed in the calculated structures of the protein with increasing extents of alkylation that is presented in Figure 4. The folded state of the apo-MT fragments can be deduced from the large differences in the alkylated species’ distribution with the same mol. eq. of NEM added (e.g. Figure 1A,B compared with Figure 1C,D). In this respect, ESI-MS is an extremely powerful tool because it allows the detection and semiquantitation of all species in solution (up to 12 in the present study) that cannot be distinguished by other means.

The first reaction of NEM with apo-MT probably occurs on a more extended conformer, the existence of which has been demonstrated in the gas phase using IM-MS under native conditions [20]. It is likely that an equilibrium exists between a number of the more collapsed and extended conformers of apo-MT in solution as well. This is especially true because the folded state is not particularly stable and able to be disrupted by the residue modification with a sufficient large moiety [21].

As further confirmation that the NEM alkylation was accurately probing the folded state of apo-MT fragments, we performed the reaction at a high enough temperature (>90°C) that should be sufficient to unfold the protein without causing precipitation. In Figure 3, it is clear that apo-βMT was unfolded, as indicated by the normal distribution of the alkylated species. This occurred without the need for chemical denaturants in solution that could potentially change the alkylation reaction itself and cause differential modification profiles that do not reflect the folded state of the protein. While this is unlikely, the high temperature experiment confirms that the protein conformation is the driving force governing the reaction profile with NEM.

### Denaturant-induced unfolding: comparison between the isolated domains of apo-MT and to model proteins

In the present study, the isolated β- and α-domain fragments of MT1a unfolded at less than 1 M and slightly more than 1 M GdmCl, respectively. This is in contrast with the most proteins with more defined secondary structures, which have been shown to unfold at GdmCl concentrations as low as 2 M, but typically between 4 and 6 M [39–41]. GdmCl was chosen as it typically unfolds proteins at lower concentrations when compared with urea denaturants and was previously used in kinetic experiments involving MT [33,42]. It has been shown that even in small proteins, transition states can be formed from non-native interactions in the presence of denaturants [43], but this was not observed for MT. It should be noted that the fragments did not unfold gradually, as indicated by the abrupt and dramatic change in the mass spectra. These results provide strong evidence that apo-MTs should not strictly be defined as intrinsically disordered proteins, which typically exhibit a linear unfolding curve as the molten globular state gradually changes with increasing denaturant concentration [44]. Thus, the unfolding of apo-MTs at a low denaturant concentration suggests that the structure is globular but is not stabilized to a significant degree. In addition, the results presented here suggest that the isolated α-domain fragment is stabilized to a more significant degree than the isolated β-domain, in accordance with the results of recent studies [22]. With respect to the full-length protein, it should be noted that the two-domain classification is based on the metal saturated structure; the distinction between domains in the apo-form is strictly for classification purposes, referring to the N- and C-terminal sections of the peptide that form the distinct dumbbell structure upon metalation. In apo-MTs the domains probably do not fold independently, but because of their divergent metal-binding behavior, the study of the isolated domains allows the examination of the mechanisms underpinning metal selectivity as well as binding affinities and mechanisms.

### Biological significance

Although this is an *in vitro* study, it is important to place the results in a biological context. Protein misfolding is associated with many diseases [45], and protein folding is a fundamental process that allows enzymes to adopt specific conformations required for catalysis or other functions. The biological functions of MT remain somewhat unclear, as knockout studies produce viable mice that are sensitive to metal exposure [46,47] and exhibit a variety of interesting but non-lethal phenotypes [48–51]. It has been proposed that in addition to metal homeostasis regulation, MT is involved in cellular redox processes due to its abundance of redox active thiols [52–54]. Recent studies have shown that the β-domain preferentially forms oxidative dimers [19], suggesting that it is more likely involved in the cellular redox processes as well. The results presented here show that the β-domain is more loosely defined, and its structure can be disrupted more easily than that of the α-domain. This suggests that the thiols are more accessible for redox reactions, as they are not strictly buried within the folded structure.

## Conclusion

We demonstrated that the isolated domain fragments of apo-MT1a are folded under native conditions and unfolded between 1 and 2 M GdmCl and at high temperature (94°C). This is in contrast with the classical description of apo-MTs being unstructured prior to metalation. We show that the β-domain is less stably folded than the α-domain, as it is more readily disrupted by the presence of denaturants. This provides further evidence for the utility of the residue modification method coupled with ESI-MS for probing the folded state of proteins lacking secondary structural features, which precludes spectroscopic characterization. These findings have important implications for the starting point of the metalation pathways of MT, suggesting a templated structure for metal binding as opposed to a random configuration. In addition, the more effective burial of cysteinyl thiols in the α-domain implies that the β-domain may be more involved in the cellular redox cycle function of MTs.

## Abbreviations

GdmCl: guanidinium chloride
IM: ion-mobility
MT: metallothionein
NEM: *n*-ethylmaleimide
TCEP: tris(2-carboxyethyl)phosphine
